# Comprehensive global collaboration in the care of 1182 pediatric oncology patients over 12 years: The Iraqi–Italian experience

**DOI:** 10.1002/cam4.4892

**Published:** 2022-06-03

**Authors:** Mazin Faisal Al‐Jadiry, Stefania Uccini, Anna Maria Testi, Maria Luisa Moleti, Adil Rabeea Alsaadawi, Amir Fadhil Al‐Darraji, Raghad Majid Al‐Saeed, Safaa A. Faraj Al‐Badri, Ahmed Hatem Sabhan, Hasanein Habeeb Ghali, Samaher Abdulrazzaq Fadhil, Wisam Majeed Abed, Najiha Ahmed Ameen, Yasir Saadoon Abed, Fawaz Salim Yousif, Aseel Rashid Abed, Hanadi Munaf Hussein, Ahmed Mudhafar Shkara, Alfonso Piciocchi, Sara Mohamed, Luigi Ruco, Ibrahim Qaddoumi, Salma Abbas Al‐Hadad

**Affiliations:** ^1^ Department of Pediatrics, College of Medicine University of Baghdad, Oncology Unit‐Children Welfare Teaching Hospital‐Medical City Baghdad Iraq; ^2^ Clinical and Molecular Medicine Sapienza University Rome Italy; ^3^ Hematology, Department of Translational and Precision Medicine Sapienza University Rome Italy; ^4^ Central Teaching Laboratory Medical City Pathology Baghdad Iraq; ^5^ Oncology Unit Children Welfare Teaching Hospital‐Medical City Baghdad Iraq; ^6^ College of Medicine‐ Wasit University Children's Welfare Teaching Hospital‐Pediatric Oncology Unit, Medical City Baghdad Iraq; ^7^ Hematology Laboratory Department Children Welfare Teaching Hospital‐Medical City Baghdad Iraq; ^8^ Department of Pharmaceutics‐College of Pharmacy University of Baghdad Baghdad Iraq; ^9^ GIMEMA Data Center GIMEMA Foundation Rome Italy; ^10^ Department of Global Pediatric Medicine St. Jude Children's Research Hospital Memphis Tennessee USA

**Keywords:** cancer, children, developing countries, pathology review, pediatric oncology, pediatric pathology, teleconsultation, telemedicine

## Abstract

**Background:**

Iraq's health care system has gradually declined after several decades of wars, terrorism, and UN economic sanctions. The Oncology Unit at Children's Welfare Teaching Hospital (CWTH) in Baghdad was lacking basic facilities and support. To address this shortcoming, a humanitarian and educational partnership was established between CWTH and Sapienza University of Rome (SUR).

**Methods:**

We investigated the outcomes of 80 online and 16 onsite educational sessions and 142 teleconsultation sessions from 2006 to 2014. We also determined the outcomes of pathology reviews by SUR of 1216 tissue specimens submitted by CWTH from 2007 until 2019 for second opinions. The primary outcomes were discordance, concordance, and changes among clinical diagnoses and pathology review findings. The measures included the frequency of teleconsultation and tele‐education sessions, the topics discussed in these sessions, and the number of pathology samples requiring second opinions.

**Findings:**

A total of 500 cases were discussed via teleconsultations during the study period. The median patient age was 7 years (range, 24 days to 16·4 years), and the cases comprised 79 benign tumors, 299 leukemias, 120 lymphomas, and 97 solid tumors. The teleconsultation sessions yielded 27 diagnostic changes, 123 confirmed diagnoses, and 13 equivocal impacts. The pathology reviews by SUR were concordant for 996 (81·9%) cases, discordant for 186 (15·3%), and inconclusive for 34 (2·8%). The major cause of discordance was inadequate immunohistochemical staining. The percentage of discordance markedly decreased over time (from 40% to 10%). The cause of the improvement is multifactorial: training of two CWTH pathologists at SUR, better immunohistochemical staining, and the ongoing clinical and pathologic telemedicine activities. The partnership yielded 12 publications, six posters, and three oral presentations by CWTH investigators.

**Interpretation:**

The exchange of knowledge and expertise across continental boundaries meaningfully improved the diagnoses and management of pediatric cancer at CWTH.

## Graphical abstract

The partnership decreased the likelihood of misdiagnoses for Iraqi children with cancer and led to more informed Iraqi care providers, thereby improving the care and outcomes of pediatric oncology patients in Iraq.

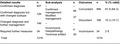

## 1 INTRODUCTION

The Children's Welfare Teaching Hospital (CWTH) is a tertiary hospital located in the Medical City Complex in Baghdad, Iraq. CWTH has a capacity of 240 beds, and the hospital is managed by the Iraqi government, with staff members who are government employees. CWTH's services are free to patients, although limited. All childhood cancers, except brain tumors, are referred from all parts of Iraq to CWTH, except for the Basra and Kurdistan governorates because of their availability of pediatric oncology units and difficulty of travel from these two regions. The CWTH Oncology Unit treats an average of 300 newly diagnosed cancer cases per year. Because of several decades of wars, terrorism, and sanctions, Iraqi health care services suffered many deficiencies that hindered optimal care for children with cancer. In addition, Iraq's difficult history has halted the natural evolution of some health care concepts, such as a multidisciplinary team approach to care (MDC). Children with cancer are among the most vulnerable groups to suffer from such challenges.

Twinning and telemedicine are proven strategies to improve care, speed the process of change, and introduce new medical concepts, including MDC, in developing countries.^1^, ^2^, ^3^, ^4^, ^5^, ^6^, ^7^, ^8^, ^9^, ^10^ Italy was one of the first Western countries to commit to supporting health care systems in Iraq. This was evident in different projects addressing the care for patients with various cancer types, such as promyelocytic leukemia,^11^ acute lymphoblastic leukemia,^12^ lymphoma,^13^, ^14^, ^15^, ^16^, ^17^, ^18^, ^19^, ^20^ Wilms tumor,^21^ and rare tumors.^22^ Here, we describe the outcomes of the telemedicine and twinning experience in pediatric oncology between Sapienza University of Rome and CWTH in Baghdad.

## 2 METHODS

### 2.1 Connecting Baghdad and Rome via telemedicine

The Simona Project is a telemedicine initiative between Italy and Iraq that is sponsored by the Italian humanitarian aid organization INTERSOS and aims to improve patient care in pediatric hematology and oncology. It was named after the kidnapping of Italian volunteer workers Simona Pari and Simona Torretta in Baghdad in September 2004 and was officially launched in February 2006. The Simona Project was renamed in 2009 to TOGETHER. However, the concept began with the involvement of pediatric hematologist Anna Maria Testi from the Hematology Center at Sapienza University of Rome.

In August 2003, Dr. Testi traveled to Baghdad to establish a collaboration with the pediatric oncology team at CWTH, evaluating the overall service for 2 weeks. On August 19, 2003, she left the United Nations (UN) building in Baghdad after meeting with Special Representative of the UN Secretary‐General to Iraq Sergio de Mello a few hours before a bombing that destroyed the building and killed Mr. de Mello. Despite nearly losing her life, she left with a promise for further collaboration. Dr. Testi later said, “This visit changed my life.” It took nearly 3 more years until February 2006, after an Iraqi team (MA and SH) visited Rome, to finalize the details of the Simona Project that led to the launching of its first teleconsultation session on April 12, 2006.

The Simona Project was supported by the European Space Agency, Policlinico Umberto I Hematology Department at Sapienza University of Rome, Telbios telemedicine company, and Italian Ministry of Foreign Affairs. The Simona Project provided one telemedicine station located at CWTH that included outdoor equipment (e.g., antenna and radio frequency transceiver unit) and indoor equipment (e.g., modem and videoconference facilities). A similar unit was also located at the Policlinico Umberto I Hematology Department at Sapienza University of Rome.

### 2.2 Teleconsultation sessions

Over an 8‐year period, 142 teleconsultation sessions were held, and 500 cases were discussed (Table 1). The team at CWTH prepared challenging cases requiring discussion with data exchange forms (DEFs) to specify patient‐related information and discuss specific questions, diagnostic equipment images (i.e., bone marrow slides, blood film slides, and diagnostic images), and all data sent to INTERSOS via email, which occurred 2 to 4 days in advance because of the poor internet connection in Baghdad. INTERSOS then forwarded the case information to the responsible advising clinicians in the Policlinico Umberto I Hematology Department at Sapienza University of Rome. The information was then reviewed, and preparations were made for live videoconference sessions. After completely discussing all of the cases, the DEFs were updated to include the outcomes (i.e., confirmed diagnoses, changed diagnoses, modified treatment strategies, problem‐solving, or further analyses) of the teleconsultation sessions.

**TABLE 1:** Teleconsultation and tele‐education sessions in the Simona‐TOGETHER Project

Year^a^	Teleconsultation sessions	No. of cases discussed	Tele‐education presentations
2003	^b^	0	^b^
2006	22	58	23
2007	11	39	6
2008	25	93	25
2009	19	72	6
2010	30	114	7
2011	17	68	7
2012	16	50	4
2013	^c^	0	^c^
2014	2	6	2
Total	142	500	80

^a^ Between 2003 and 2006, preparation for the Simona project was in process.

^b^ In 2003, AMT visited Iraq and presented six educational sessions over 2 weeks.

^c^ In 2013, the team from Sapienza University of Rome was present in Iraq, and ten educational sessions were held onsite.

The meetings were scheduled on a weekly basis, with modifications according to the availability of the internet and time availability of both teams. The selection of cases depended on the priority of management and the debate on the diagnosis, as determined by the Iraqi team according to the complexity of cases. The median duration of teleconsultation sessions was 2 h (range, 1 h to 3.5 h) depending on the number of cases and the presence of educational lectures. The median number of cases discussed in each session was 7 (range, 1–11). In addition, email exchanges were used for urgent cases or to expand upon the teleconsultation sessions.

### 2.3 Tele‐education

Over the 8‐year period, 80 educational sessions were held (Table 1). PowerPoint presentations related to the scientific topics chosen were prepared in Rome and/or Baghdad according to the needs in Iraq, which were related to the general overview of a specific disease, its management or complications, and histologic descriptions of pathology samples that required second opinions. Pediatric cancer treatment regimens and supportive care measures were also discussed. Modern therapeutic programs for the management of various cancers in Iraqi children were tailored and adapted to the local and social conditions. In 2013, the Italian team visited CWTH for 1 week and presented ten educational sessions.

### 2.4 Pathology review

Two pathology specimens were successfully transferred in person to Rome in 2006 and analyzed by the Pathology Department of Sapienza University of Rome. Therefore, the teams at CWTH and Sapienza University of Rome concluded that formalin‐fixed, paraffin‐embedded tissue blocks could be shipped from Baghdad to Rome while keeping their physical properties intact. Consequently, a meeting in Rome with two Italian pathologists (SU and LR) occurred in January 2007 to develop a protocol for future pathology reviews. The first samples were shipped in May 2007, and then clusters of five to ten samples per month (≤10 samples/month) were delivered to Rome on a regular basis. In Rome, after receiving the DEFs from Baghdad, a full analysis of the tissue morphology, immunohistochemical (IHC) staining, and genetic analyses (according to specific needs) was performed. Formal pathology reports were then sent to Baghdad by email, and interesting or concerning cases were discussed during live tele‐education sessions. The outcomes of the second opinions were decided after discussion among the pathologists in Iraq and Italy. Accordingly, we classified the outcomes of the formal pathology reports generated from May 2007 until October 2019 as follows: (i) concordance, i.e., retaining the original disease management strategy after either confirmed diagnosis or confirmed diagnosis with a changed subtype; (ii) discordance, i.e., changing or modifying the disease management strategy after either a changed diagnosis or changed subtype; or (iii) inconclusive, i.e., inconclusive histopathology findings due to either inadequate sample collection or technical artifacts.

### 2.5 Exchange visits

Exchange visits occurred between both teams and were determined according to the needs at CWTH and planned via email and videoconference sessions.

### 2.6 Statistical analysis and ethical review

We compiled and analyzed the data presented in this study by examining all of the DEFs, email exchanges, and project reports generated during the 8‐year study period and the pathology reports generated during the 12‐year study period. Because all data collected in this study comprised nominal categorical data, the frequencies and proportions (%) are presented. This study was approved by the Ethical Review Committee at CWTH.

## 3 RESULTS

### 3.1 Teleconsultation and email exchanges

Teleconsultation via video conferences was supported by email exchange if any technical obstacles or urgent cases required immediate attention. The first teleconsultation session occurred April 12, 2006, and the last session occurred March 20, 2014. During this period, 142 live sessions discussed 500 cases, of which 282 new patients and 218 recurrent patients were included (Tables 1 and 2). Some patient cases were discussed in more than one teleconsultation session. Additionally, 95 patient cases were urgently discussed via email exchange (30 new patients and 65 patients with recurrent disease). The consultations were related to reviews of blood film/bone marrow slides, discussions about management plans, or explanations of changes in diagnoses after re‐evaluation of pathology specimens.

**TABLE 2:** Results of teleconsultation sessions and email exchanges

Data	Outcome	Teleconsultations	Emails	Total Exchanges
Peripheral smears or bone marrow slides	Confirmed diagnosis	119	4	123
	Changed diagnosis	25	2	27
	Equivocal	10	3	13
Interpretation of IHC findings in pathology review		48	12	60
Clinical management	Management strategy impacts	284	74	358
	Further discussion and analysis	14	0	14
Total		500	95	595

Abbreviation: IHC, immunohistochemistry.

A total of 595 cases were discussed (Table 2). The median patient age was 7 years (range, 24 days to 16.4 years); 374 patients were boys, and 221 patients were girls. The cases discussed included benign tumors (*n* = 79), leukemias (*n* = 299), lymphomas (*n* = 120), and solid tumors (*n* = 97). The median number of cases per session discussed via teleconsultation was 7 (range, 1–11 cases). The discussions were categorized according to three themes: blood smears/bone marrow slides, clinical management strategies, and IHC findings. Review of peripheral smears or bone marrow slides occurred in 163 cases (Table 2). This resulted in diagnostic changes (*n* = 27), confirmed diagnoses (*n* = 123), and equivocal impacts (*n* = 13). The characteristics of the 27 cases with diagnostic changes are defined in Table 3. Clinical management strategies were discussed in 372 cases (Table 2). Of these, 358 discussions included the impacts on the management strategies, and 14 required further discussion and analysis by the Italian team. The interpretation of IHC findings in the pathology review cases that resulted in changed diagnoses were discussed in 60 cases (Table 2).

**TABLE 3:** Major changes in diagnoses via teleconsultation or email exchanges (*n* = 27 patients)

Patient no.	CWTH diagnosis	Sapienza University diagnosis
1	ALL	AML
2	AL	AML
3	APL	AML‐M2
4	Lymphoblastic lymphoma	Hodgkin Lymphoma
5	Lymphoblastic lymphoma	Poorly differentiated neuroblastic tumor
6	AL	ALL
7	AL	ALL
8	Rhabdomyosarcoma	AML
9	APL	AML
10	Malignant fibrous histiocytoma	Soft tissue sarcoma
11	Lymphoblastic lymphoma	AML
12	APL	AML
13	AL	ALL
14	APL	Anemia
15	Acute leukemia	ALL
16	AL	AML
17	AL	ALL
18	ALL	AML
19	ALL	Neuroblastoma
20	NHL	Neuroblastoma
21	APL	AML
22	APL	AML‐M5
23	AL	ALL
24	NHL	AML
25	AL	AML
26	ALL	T‐cell ALL
27	Neuroblastoma	AML

Abbreviations: AL, acute leukemia without specific type; ALL, acute lymphocytic leukemia; AML, acute myelocytic leukemia; APL, acute promyelocytic leukemia; AUL, acute undifferentiated leukemia; CWTH, Children Welfare Teaching Hospital; LL, lymphoblastic lymphoma; NHL, non‐Hodgkin lymphoma.

### 3.2 Tele‐education

In 2003, an Italian team member (AMT) provided an introductory intensive course of lectures (*n* = 6) over 2 weeks, covering different types of leukemia. After the telemedicine project was initiated, the tele‐education sessions consisted of 80 online and ten onsite lectures on various topics, including lymphomas (*n* = 30), leukemias (*n* = 18), benign hematology (*n* = 10), oncologic emergency/infections (*n* = 16), pathology topics (*n* = 10), and solid tumors (*n* = 6).

### 3.3 Exchange visits

Although the core aim of the initiative between Rome and Baghdad was telemedicine, which is second‐best to in‐person consultations,^23^ both teams realized that occasional in‐person meetings were needed. Therefore, exchange visits were an important component of the Simona Project. In addition, both teams planned meetings at international conferences. Table S1 summarizes all of the exchange visits by both teams and meetings at international conferences.

### 3.4 Pathology review

Second opinions on pathologic findings comprised another major component of this initiative. Over a 12‐year period (May 2007 to October 2019), 1220 pathologic samples were reviewed. Four of these samples were excluded from review because one was lost during shipping, one was incorrectly sent, and two had autolysis present but were later replaced with properly prepared paraffin‐embedded blocks. Therefore, the total number of samples was 1216, which were obtained from 1182 patients. Of these patients, 734 were male and 448 were female, with a median age of 66 months (range, 2–191 months). Some patients had two pathologic samples that were reviewed (*n* = 26), and some had three samples reviewed (*n* = 4) (Table S2). The reasons for these repeated sample reviews included insufficient previous samples (*n* = 9), suspicion of relapse (*n* = 20), new lesions (*n* = 3), residual lesion (*n* = 1), and follow‐up (*n* = 1). Repeated biopsies of the initial site occurred in 23 patients, and those of a different site occurred in 11 patients.

The diagnostic pathology performed at CWTH relied on morphology for 929 patients, and IHC analysis was used in only 287 cases (23·6%). The Iraqi team built local expertise by ensuring that dedicated pathologists examined most of the samples. Two pathologists examined 92·4% of the cases. Biopsy types comprised excisional (*n* = 404), incisional (*n* = 428), lymph node (*n* = 363), tru‐cut (*n* = 10), and bone marrow (*n* = 11) tissues. Lymph nodes were the most numerous (*n* = 363) tissue type, followed by renal (*n* = 152) and soft tissues (*n* = 108), as shown in Table S3.

Second opinions had a profound effect on patient care at CWTH. Disease management strategies were markedly altered due to changes in diagnoses for 15·3% of patients (Table 4). Most of the cases with discordance comprised lymphoma or soft‐tissue tumors (Table S4). The second opinion reviews considerably improved the expertise of the local dedicated pathologists. Indeed, discordant diagnoses dropped from 27·6% for the first 25% of samples reviewed (*n* = 304) to 7·6% for the last 25% of samples reviewed (*n* = 304) (Table 5). The Iraqi team further analyzed the discordance in the first 100 samples submitted to Rome and compared it with that of the last 100 samples. The difference was striking (40% vs. 10%) (Table 5). Second opinions were also useful for improving the capacity of interpretation of IHC analyses. Specifically, discordant interpretations declined from 17·6% for the first 25% of samples (*n* = 304) to 12·1% for the last 25% of samples (*n* = 304).

**TABLE 4:** Discordance rates in samples submitted for second pathologic review to Sapienza University of Rome

Detailed results	*n*	Sub‐analysis	*n*	Outcomes	*n*	% (% valid)
Confirmed diagnosis	907	–	–	Concordant	996	81.9 (84.3)
Confirmed diagnosis but with changed subtype	126	Confirmed management	89			
		Modified management	37	Discordant	186	15.3 (15.7)
Changed diagnosis and further management	149	–	–			
Required further measures	34	Inconclusive histopathology	4	Inconclusive	34	2.8
		Technical artifact	30			
Total	1216				1216	

**TABLE 5:** Chronology of sample discordance

Group no.^a^	No.	Concordant	Discordant	Inconclusive	%
1	304	210	84	10	27.6
2	304	245	49	10	16.1
3	304	267	30	7	9.9
4	304	274	23	7	7.6
Total	1216	996	186	34	15.3
First 100 samples	100	57	40	3	40
Last 100 samples	100	86	10	4	10

^a^ Groups were assigned as quartiles in the time of analysis for groups of 304 cases, in which Group 1 was the earliest 304 cases diagnosed and Group 4 was the last 304 cases diagnosed.

### 3.5 Publications

Although this comprehensive initiative to improve the outcomes of Iraqi children specifically focused on patient care and building local capacity, it also promoted research at CWTH, culminating in several peer‐reviewed publications. In addition to 12 publications produced by the Simona‐TOGETHER Project,^11^, ^12^, ^13^, ^14^, ^15^, ^16^, ^17^, ^18^, ^19^, ^20^, ^21^, ^22^ researchers at CWTH presented six posters and three oral presentations at international and regional conferences. Such research outcomes are important for the CWTH clinical investigators' professional development and acknowledgement at both regional and international levels.

## 4 DISCUSSION

To our knowledge, this is the largest and most comprehensive telemedicine experience in pediatric oncology that supported patient care, education, research, and local capacity building. Because pediatric care in the Oncology Unit at CWTH continues to thrive even after ending the telemedicine experience (live sessions ended in 2014 and pathology activities ended in October 2019), the fundamental outcome of this telemedicine initiative was sustainability. We believe that this success was due to the will and dedication of both academic institutions, in addition to the support of such stakeholders as the Iraqi Ministry of Health, INTERSOS, Telbios, and the Italian Ministry of Foreign Affairs.

Telemedicine improves the care of patients with pediatric cancer,^8^, ^9^, ^10^ including complex cases such as brain tumors^5^, ^6^ and retinoblastoma.^7^ The Iraqi–Italian experience is unique among previous telemedicine initiatives because of the large number of patients discussed, the diverse types of pediatric neoplasms covered, and its emphasis on building local capacity and education. This was evident in the drop in pathology IHC discordance from 17·6% at the start of the experience to 12·1% at the end of the study. The decrease in morphology discordance was more striking—27·6% at the start to 7·6% at the end. This improvement is attributed to pathology consultation, continuous virtual training, improvement in the technique of staining, and possibly to the onsite training of two Iraqi pathologists. Others have demonstrated similar decreased discordance rates over time,^5^, ^7^, ^8^, ^9^ further demonstrating the role of telemedicine in improving local capacity and knowledge. This was evident in the decreased email exchanges over the last few years (2014–2019) of the initiative as the collective local team knowledge improved and the need for second opinions decreased. Most of the diagnostic discordances were caused by poor IHC staining techniques affecting the quality of the histologic samples and/or by a lack of updated knowledge of new tumor classifications.

This lack of knowledge was expected because of the long academic isolation that the Iraqi colleagues suffered due to sanctions, wars, and civil unrest. Thus, the tele‐education sessions and training visits for the Iraqi colleagues were crucial to bridge this knowledge gap. Sending pathologic materials overseas is costly and time consuming and causes delays in pathologic reporting. One solution for this is digital telepathology.^24^, ^25^ Recent technical advancements allow transformation of histologic specimens mounted on glass slides into digital files that can be sent to any location in real time via the internet. Furthermore, artificial intelligence algorithms can now interpret the images contained in the files and suggest diagnoses.^26^ A great advantage of these new techniques is their ability to facilitate the exchange of interpersonal opinions of histopathologic diagnoses. Moreover, once these artificial intelligence applications are fully developed and considered reliable, they will most likely help pathologists reach correct diagnoses. This will be a major advance in bridging pediatric oncology knowledge gaps between continents.

The Italian colleagues are seeking ways to expand upon this experience. One example is improving access to molecular pathology. Targeted therapy is one of the most beneficial advances in medical oncology. However, its use requires accurate histologic diagnoses with molecular characterization of tumors.^27^ Medical companies are now developing instruments that are easy to use and can provide full molecular characterizations of tumors. We believe that these new technologies will become used in Iraqi pathology laboratories soon, thereby further reducing the knowledge gap between continents.

Another important outcome of this experience is improved patient outcomes, evidenced by decreasing mortality, toxicities, infections, treatment abandonment, and relapse rates.^11^, ^12^, ^13^, ^18^ A striking example of such improved outcomes was a decreased rate of early death from 12% to 6% for patients with acute lymphoblastic leukemia by the introduction of pre‐phase steroid treatment.^12^ Another excellent example is evident from cases in which initial local diagnoses of malignant tumors were found to be benign (*n* = 31) after telemedicine and pathology review. These major changes in diagnoses spared these patients from unnecessary toxic therapies and conserved precious and limited local resources that could be used for other patients (Table S5). In addition, the diagnoses of 27 cases were changed through this cooperation, which had profound implications on the final treatment regimens the patients received. Another impressive example of the improved patient outcomes afforded by the Simona‐TOGETHER Project was a reduced induction mortality rate in patients with acute promyelocytic leukemia from 95% to 5% through telemedicine, pathology reviews, and improved access to therapies.^11^

To illustrate the relevance and impact of this initiative, we share an example of a case in which cooperation saved the life of a young child. A 2‐month‐old boy with acute lymphoblastic leukemia had no detectable veins. The team in Italy advised CWTH providers to use subcutaneous injection of cytarabine—a protocol developed in the early 1980s.^28^, ^29^ The team at CWTH used subcutaneous cytarabine for 2 months to induce remission before the patient received full treatment. This patient is now 7 years post treatment and enjoys an excellent quality of life. In a country suffering four decades of wars, invasion, civil war, and terrorism, this would not have been possible without colleagues crossing continental boundaries to exchange knowledge and better humanity.

Over time, the diagnostic tools improved, IHC started to be done on a regular basis in the public and private laboratories, flow cytometry was introduced to further classify the types of leukemia, and the oncology team gained substantial experience in managing different types of cancer.

The cultural impact on local health care systems is another outcome of such initiatives but is difficult to quantify. These initiatives introduce important concepts for optimal patient care, such as second opinions, protocol adherence, toxicity vigilance, therapy modifications, up‐to‐date knowledge of the literature, and the concepts of sub‐specialization and MDC. These cultural changes become integral to the local systems and continue long after the telemedicine experience ends. These changes also affect patients, which was evident by the decreased treatment abandonment rates for various cancers treated at CWTH. The four decades of strife in Iraq affected not only its health care infrastructure but also patient trust in these systems. Because patients at CWTH were informed about the consultation initiative with Italy, many parents felt that they could trust the treatment plan provided by the local team.^5^, ^6^, ^7^, ^8^

Both teams felt that an adequate threshold of local capacity in Iraq was achieved. However, there is still access to pathologic consultation when needed. The scientific and clinical collaboration between CWTH and SUR is still ongoing, with exchange of clinical cases, review of immunophenotype and radiological results, design of new protocols for different pediatric hematologic diseases, and statistical data analysis. The team in Iraq is also diversifying in second clinical and pathological review with St. Jude Children's Research Hospital (St. Jude), American University of Beirut (AUBMC) in Lebanon and Sidra Medicine in Qatar.

This experience primarily addressed inequalities in leukemia, lymphoma, and Wilms tumor. To address disparities in the treatment of other childhood cancers in Iraq, the team at CWTH recently launched an MDC service for retinoblastoma in collaboration with St. Jude and King Hussein Cancer Center (KHCC) in Amman, Jordan. It also launched an MDC service for brain tumors in collaboration with St. Jude. Another effort with Sidra medicine is reviewing pathology materials and providing MDC services for Wilms tumor. The impact of these new initiatives on brain tumors, retinoblastoma, and Wilms tumor is yet to be seen, but we are hopeful that it will be similar to the one described here. Collaboration with St. Jude through the POEM region the Iraqi team received access to different supportive sites, including Jordan (KHCC) and Lebanon (AUBMC). Currently, Iraqi team in CWTH are prospectively discussing every CNS tumor case with St. Jude and every retinoblastoma case with KHCC and St. Jude. The impact on promoting research at CWTH was also outstanding, considering the challenges facing the Iraqi colleagues at every level of academia and patient care. This was evident in the number of publications authored by CWTH providers (*n* = 12) and the locally grown research culture.

In conclusion, when politicians and leaders use technology to send intercontinental missiles and warplanes, doctors can send intercontinental knowledge and medical care. We hope that these politicians and leaders can learn something from pediatric oncologists.

## AUTHORS CONTRIBUTION

Study conception and design: Mazin Faisal Al‐Jadiry, Stefania Uccini, Anna Maria Testi, Maria Luisa Moleti, Luigi Ruco, Ibrahim Qaddoumi and Salma Abbas Al‐Hadad. Data collection: Mazin Faisal Al‐Jadiry, Amir Fadhil Al‐Darraji, Raghad Majid Al‐Saeed, Safaa A. Faraj Al‐Badri, Ahmed Hatem Sabhan, Hasanein Habeeb Ghali, Samaher Abdulrazzaq Fadhil, Wisam Majeed Abed, Najiha Ahmed Ameen, Yasir Saadoon Abed, Fawaz Salim Yousif, Aseel Rashid Abed, Hanadi Munaf Hussein, Ahmed Mudhafar Shkara, Wisam Majeed Abed, Najiha Ahmed Ameen, Alfonso Piciocchi and Sara Mohamed. Analysis and interpretation of results: Mazin Faisal Al‐Jadiry, Stefania Uccini, Anna Maria Testi, Maria Luisa Moleti, Luigi Ruco, Adil Rabeea Alsaadawi, Safaa A. Faraj Al‐Badri, Hasanein Habeeb Ghali, Ibrahim Qaddoumi and Salma Abbas Al‐Hadad. Draft manuscript preparation: Mazin Faisal Al‐Jadiry, Stefania Uccini, Anna Maria Testi, Maria Luisa Moleti, Luigi Ruco, Ibrahim Qaddoumi and Salma Abbas Al‐Hadad. All authors reviewed the results and approved the final version of the manuscript.

## CONFLICT OF INTEREST

The authors have declared that no conflict of interest exists.

## ETHICS STATEMENT

The Institutional Ethics Committee at Children Welfare Teaching Hospital‐Medical City Baghdad has approved the study protocol, which follows the Declaration of Helsinki.

## Supporting information


Table S1‐S5

## Data Availability

The authors were unable to find a valid data repository for the data used in this study. These data are available from Mazin Al‐Jadiry at Children Welfare Teaching Hospital‐Medical City Baghdad.
