# Supplementary material for: Comprehensive global collaboration in the care of 1182 pediatric oncology patients over 12 years: The Iraqi–Italian experience
Source: Cancer Med. 2022 Jun 3;12(1):256–65. doi: 10.1002/cam4.4892 (PMC9844594; doi:10.1002/cam4.4892)
Supplement: Supplementary file 1 — Table S1‐S5 [file CAM4-12-256-s001.docx]

**Supplementary Tables**

| **Date** | **Communication**  **methods and events** | **Topic(s) discussed** | **Impact/outcome** |
| --- | --- | --- | --- |
| August 2003 | AMT visit to CWTH | Overall assessment | Telemedicine project |
| February 2006 | CWTH group visit to SUR, 3-day workshop | Telemedicine program | Launching of the live meetings in April 2006 |
| September 2006 | CWTH and SUR teams met in Amman for 5-day workshop | Donation of drugs | Mitigation of supply shortage of chemotherapies |
| November 2006 | Hematopathologist training for 1 month in hematology laboratory at SUR | Improved IHC staining | Provided IHC staining materials |
| January 2007 | CWTH group visit at SUR for 6 weeks | Attending conference and training | Improved practice  and orientation in oncology statistics |
| October 2007 | CWTH physician met with INTERSOS representative in UK | Details of telemedicine program | Solved technical and logistic problems |
| November 2007 | Training of 1 CWTH oncologist | Management of emergencies | Improved practice in supportive care |
| November 2007 | CWTH group visit to SUR to attend conference | Telemedicine collaboration | Official signature of both parties for pathology review |
| September 2009 | Attending APL conference | Outcome of CWTH APL patients | Revision and update of modified APL protocol |
| November 2012 | Training of 1 CWTH pathologist for 1 month | Improve morphology and IHC staining | Reduction in discordant results for pathology reviews |
| March 2013 | SUR team visited CWTH | Orientation and telemedicine program progression | Failed to convince the government to change the system of pathology procedures and logistics |
| September 2013 | APL symposium | Outcomes of CWTH patients with APL | Presentation of APL outcomes |
| May 2015 | CWTH physician visited SUR | Draft of lymphoma manuscript | Publication |
| December 2015 | CWTH and SUR investigators met at SIOP in Orlando | Collaboration on publications | Publication |
| September 2017 | APL symposium | Follow-up of CWTH patients with APL | Presentation of APL outcomes |
| October 2019 | CWTH group pass-by visit to SUR during SIOP meeting | ALL 17-year outcomes | Poster presentation at SIOP |
| January 2019 | Training of 1 CWTH pathologist for 1 month | Morphology and IHC staining of lymphoma cases | Decreased discordance in pathology |
| AMT=Anna Maria Testi. APL=acute promyelocytic leukemia. CWTH=Children’s Welfare Teaching Hospital. IHC=immunohistochemistry. SIOP=International Society of Pediatric Oncology. SUR=Sapienza University of Rome. UK=United Kingdom. | | | |
| ***Table S1:* Details of exchange visits and in-person meetings** | | | |

| **Reason for subsequent review** | **Initial anatomic site** | **No.** | **Final anatomic site** | **SUR diagnosis of first sample sent from CWTH** | **SUR diagnosis of recurrent samples** | **Tumor type** |
| --- | --- | --- | --- | --- | --- | --- |
| Diagnosis | Same | 7 | Cervical LN | Reactive LAP | Reactive LAP | Benign |
|  |  |  | Cervical LN | Reactive LAP | HL-NLP | Malignancy |
|  |  |  | Cervical LN | Reactive LAP | Reactive LAP | Benign |
|  |  |  | Liver | Inconclusive-technical artifact | Inconclusive-technical artifact | Inconclusive |
|  |  |  | Vagina | Inconclusive-technical artifact | Inconclusive-technical artifact | Inconclusive |
|  |  |  | Vagina | Inconclusive-technical artifact | GCT-YST | Malignancy |
|  |  |  | Kidney | Multicystic nephroma | WT-blastema | Malignancy |
|  | Different | 2 | SC LN | Inconclusive-technical artifact | Inconclusive-technical artifact | Inconclusive |
|  |  |  | SC LN | Inconclusive-technical artifact | Peripheral T-cell lymphoblastic lymphoma | Malignancy |
| Relapse | Same | 14 | Cervical LN | T-cell lymphoblastic lymphoma | Reactive LAP | Benign |
|  |  |  | Cervical LN | HL-MC | Reactive LAP | Benign |
|  |  |  | Cervical LN | HL-MC | Reactive LAP | Benign |
|  |  |  | Cervical LN | HL-MC | Reactive LAP | Benign |
|  |  |  | Cervical LN | HL-MC | Reactive LAP | Benign |
|  |  |  | Cervical LN | Reactive LAP | HL-NLP | Relapse |
|  |  |  | Cervical LN | Lymphoma-HL-NS | HL-MC | Relapse |
|  |  |  | Cervical LN | HL-MC | Lymphoma-HL-MC | Relapse |
|  |  |  | Abdominal LN | Burkitt lymphoma | Reactive LAP | Benign |
|  |  |  | Abdominal LN | Fibrous synovial sarcoma | Residual fibrosis | Benign |
|  |  |  | Sacro coccyx | GCT-YST | Necrotic reaction | Benign |
|  |  |  | Sacro coccyx | GCT-YST | Mature cystic teratoma | Benign |
|  |  |  | Bladder | RMS-embryonal | Inflammatory reaction | Benign |
|  |  |  | Tonsil | Burkitt lymphoma | Inflammatory condition | Benign |
|  | Different | 6 | Abdominal LN | DLBCL | Reactive LAP | Benign |
|  |  |  | Abdominal LN | HL-NS | HL-NS | Relapse |
|  |  |  | Abdominal LN | HL-MC | HL | Relapse |
|  |  |  | Cervical LN | HL-NS | Reactive LAP | Benign |
|  |  |  | Mediastinum | HL-MC | HL-MC | Relapse |
|  |  |  | Postnasal | HL-NS | Inflammatory condition | Relapse |
| New lesion | Different | 3 | Cervical LN | Xanthogranulomatous pyelonephritis | HL-NS | Malignancy |
|  |  |  | Left Ilium | HL-MC | Chondromyxoid fibroma | Benign |
|  |  |  | Tibia | Burkitt lymphoma | Osteomyelitis | Benign |
| Follow-up | Same | 1 | Kidneys | WT-blastema | WT-post chemotherapy | Benign |
| Residual | Same | 1 | Bowel | Burkitt lymphoma | Inflammation | Benign |
| DBLCL=diffuse large B-cell lymphoma. GCT=germ cell tumor. HL=Hodgkin lymphoma. LAP=lymphadenopathy. NHL=non-Hodgkin lymphoma. LN=lymph node. MC=mixed cell. NLP=nodular lymphocyte predominant. NS=nodular sclerosis. RMS=rhabdomyosarcoma. SC=supraclavicular. WT=Wilms tumor. YST=yolk sac. tumor. | | | | | | |
| ***Table S2:* Details of 34 subsequently reviewed samples from 30 patients (n = 30 second samples and n = 4 third samples)** | | | | | | |

| **Site/group** | | | | | | | **No.** |  |
| --- | --- | --- | --- | --- | --- | --- | --- | --- |
| Lymphatics: cervical (n = 276), abdominal (n = 25), axillary (n = 23),  supraclavicular (n = 17), inguinal (n = 14), para-auricular (n = 5), and thigh (n = 3) | | | | | | | 363 |  |
| Kidney | | | | | | | 152 |  |
| Gastrointestinal tract: bowel/ampulla of vater (n = 1), esophagus (n = 1), rectum (n = 3), and stomach (n = 1) | | | | | | | 106 |  |
| Soft tissue | | | | | | | 108 |  |
| Bone: femur (n = 13), tibia (n = 8), fibula (n = 6), humerus (n = 4), rib (n = 4), scapula (n = 3), ilium (n = 2), calcanium (n = 1), clavicle (n = 1), frontal (n = 1), mastoid (n = 1), and sternum (n = 1) | | | | | | | 45 |  |
| Abdominal cavity | | | | | | | 47 |  |
| Adrenal gland | | | | | | | 46 |  |
| Eye/eyelid | | | | | | | 43 |  |
| Oronasopharynx | | | | | | | 41 |  |
| Mediastinum | | | | | | | 34 |  |
| Jaw | | | | | | | 29 |  |
| Ovary | | | | | | | 24 |  |
| Pelvic/peritoneal | | | | | | | 22 |  |
| Sacrococcyx | | | | | | | 22 |  |
| Skin | | | | | | | 20 |  |
| Retroperitoneal mass | | | | | | | 21 |  |
| Liver | | | | | | | 19 |  |
| Spinal/paraspinal mass | | | | | | | 15 |  |
| Testis | | | | | | | 16 |  |
| Bone marrow | | | | | | | 11 |  |
| Lung/pleura | | | | | | | 9 |  |
| Uterus and vagina | | | | | | | 8 |  |
| Urinary bladder | | | | | | | 6 |  |
| Pancreas | | | | | | | 2 |  |
| Parotid gland | | | | | | | 2 |  |
| Spleen | | | | | | | 2 |  |
| Pericardium | | | | | | | 1 |  |
| Thyroid | | | | | | | 1 |  |
| Brain | | | | | | | 1 |  |
| Total | | | | | | | 1216 |  |
| SUR, Sapienza University of Rome. | | | | | | |  |  |
| ***Table S3:* Anatomic sites of samples submitted for second pathology review at SUR** | | | | | | |  |  |
| **Tumor site/type or disorder** | **No.** | **SUR-diagnosed diseases** | **No.** | **Concordant** | **Discordant** | **Discordance error (%)** | | |
| Adrenal gland tumors | 10 | Adrenocortical adenoma | 4 | 3 | 1 | 20·0 | | |
|  |  | Adrenocortical carcinoma | 5 | 4 | 1 |  |  |  |
|  |  | Pheochromocytoma | 1 | 1 | 0 |  |  |  |
| Bone-forming tumors | 10 | Osteogenic sarcoma | 9 | 9 | 0 | 10·0 | | |
|  |  | Osteoblastoma | 1 | 0 | 1 |  |  |  |
| EWING-PNET | 71 | EWING-PNET | 71 | 61 | 10 | 14·0 | | |
| Germ cell tumors | 70 | Malignant germ cell tumor | 52 | 50 | 2 | 8·6 | | |
|  |  | Mature cystic teratoma | 18 | 14 | 4 |  |  |  |
| Histiocytic disorder | 28 | Erdheim–Chester disease | 1 | 0 | 1 | 28·6 | | |
|  |  | HLH | 1 | 0 | 1 |  |  |  |
|  |  | LCH | 20 | 18 | 2 |  |  |  |
|  |  | Rosai–Dorfman | 6 | 2 | 4 |  |  |  |
| Inflammatory reaction | 95 | Others | 25 | 20 | 5 | 13·7 | | |
|  |  | Xanthogranulomatous process | 6 | 6 | 0 |  |  |  |
|  |  | Reactive LAP | 64 | 56 | 8 |  |  |  |
| Inflammatory disease | 7 | Inflammatory/hyperplastic diseases | 2 | 1 | 1 | 42·8 | | |
|  |  | Tuberculosis | 5 | 3 | 2 |  |  |  |
| Leukemia | 8 | Leukemia | 8 | 4 | 4 | 50·0 | | |
| Liver tumors | 13 | Hepatocellular carcinoma | 1 | 0 | 1 | 7·7 | | |
|  |  | Hepatoblastoma | 12 | 12 | 0 |  |  |  |
| Lymphomas | 439 | Hodgkin lymphoma | 191 | 174 | 17 | 12·3 | | |
|  |  | NHL-B-cell | 197 | 177 | 20 |  |  |  |
|  |  | NHL-T-cell | 51 | 34 | 17 |  |  |  |
| Nasopharyngeal carcinoma | 5 | Nasopharyngeal carcinoma | 5 | 4 | 1 | 20·0 | | |
| Peripheral neuroblastic tumors | 96 | Ganglioneuroma | 11 | 8 | 3 | 13·5 | | |
|  |  | Ganglioneuroblastoma | 12 | 12 | 0 |  |  |  |
|  |  | Neuroblastoma | 73 | 63 | 10 |  |  |  |
| Retinoblastoma | 12 | Retinoblastoma | 12 | 12 | 0 | 0·0 | | |
| Renal tumors | 144 | CCSK | 11 | 9 | 2 | 13·2 | | |
|  |  | Mesoblastic nephroma | 5 | 2 | 3 |  |  |  |
|  |  | Renal cell carcinoma | 3 | 3 | 0 |  |  |  |
|  |  | Wilms tumor | 125 | 111 | 14 |  |  |  |
| Skin tumors | 5 | Skin tumors | 5 | 2 | 3 | 60·0 | | |
| Soft tissue tumors | 145 | Fibromatosis | 19 | 13 | 6 | 29·7 | | |
|  |  | Infantile fibrosarcoma | 7 | 7 | 0 |  |  |  |
|  |  | Others | 23 | 8 | 15 |  |  |  |
|  |  | PNST-benign | 3 | 2 | 1 |  |  |  |
|  |  | PNST-malignant | 8 | 0 | 8 |  |  |  |
|  |  | Rhabdomyosarcoma | 76 | 68 | 8 |  |  |  |
|  |  | Synovial sarcoma | 9 | 4 | 5 |  |  |  |
| Others | 24 | Other conditions | 24 | 19 | 5 | 20·8 | | |
| Total | 1182 |  | 1182 | 996 | 186 | 33·1 | | |
| CCSK=clear cell sarcoma of kidney. HLH=hemophagocytic lymphohistiocytosis. LAP=lymphadenopathy. LCH=Langerhans cell histiocytosis. NHL=non-Hodgkin lymphoma. PNET=peripheral nerve sheath tumor. PNST=peripheral nerve sheath tumor. | | | | | | | | |
| ***Table S4:* Concordance/discordance according to type/site of tumors** | | | | | | | | |

| **Case no.** | **Malignant cases diagnosed in Iraq** | **Changed to benign diagnoses in Rome** |
| --- | --- | --- |
| 1 | Clear cell sarcoma of Kidney | Congenital mesoblastic nephroma |
| 2 | Fibrosarcoma | Myofibromatosis |
| 3 | Fibrosarcoma | Congenital mesoblastic nephroma |
| 4 | Ganglioneuroblastoma | Ganglioneuroma |
| 5 | Ganglioneuroblastoma | Ganglioneuroma |
| 6 | GIST | Inflammatory myofibroblastic tumor |
| 7 | GIST | Inflammatory myofibroblastic tumor |
| 8 | HL | Chronic lymphadenitis with Castleman-like features |
| 9 | HL | Chronic reactive lymphadenitis |
| 10 | HL | Chronic reactive lymphadenitis |
| 11 | HL | Cystic lymphangioma |
| 12 | HL | Kikichi–Fujimoto disease |
| 13 | HL | Reactive follicular hyperplasia |
| 14 | Immature teratoma | GCT/Mature cystic teratoma |
| 15 | Immature teratoma | GCT/Mature cystic teratoma |
| 16 | Immature teratoma | GCT/Mature cystic teratoma |
| 17 | Immature teratoma | GCT/Mature cystic teratoma |
| 18 | MPNST | Infantile fibromatosis |
| 19 | Neuroblastoma | Ganglioneuroma |
| 20 | NHL | Chronic reactive lymphadenitis |
| 21 | NHL | Chronic reactive lymphadenitis |
| 22 | NHL | Inflammatory reaction |
| 23 | NHL | Inflammatory reaction |
| 24 | NHL | Reactive inflammatory process |
| 25 | Osteosarcoma | Bone tumors/Osteoblastoma |
| 26 | Osteosarcoma | Rosai Dorfman |
| 27 | RMS | Hamartoma |
| 28 | RMS | Hemangiopericytoma |
| 29 | Soft tissue sarcoma | Histiocytosis |
| 30 | Soft tissue sarcoma | Juvenile capillary hemangioma |
| 31 | WT | Renal tumors/Congenital mesoblastic nephroma |
| GCT=germ cell tumor. GIST=gastrointestinal stromal tumor. HL=Hodgkin lymphoma. NHL=non-Hodgkin lymphoma. MPNST=malignant peripheral nerve sheath tumor. RMS=rhabdomyosarcoma. WT=Wilms tumor. | | |
| ***Table S5:*** **Malignant diagnoses changed to benign diagnoses** | | |
